# Design and analysis of a mechanical ventilation system based on cams

**DOI:** 10.1016/j.heliyon.2021.e08195

**Published:** 2021-10-19

**Authors:** J. Alan Calderón Ch, Carlos Rincón, Martin Agreda, Juan José Jiménez de Cisneros

**Affiliations:** a Angewandte Nanophysik, Institut für Physik, Technische Universität Ilmenau, Ilmenau 98693, Germany; b Engineering Department, Pontificia Universidad Católica del Perú, Lima 15088, Peru

**Keywords:** Mechanical ventilation design, Low-cost mechanical ventilator, Experimental ventilation curves, Mechanical ventilation mathematical model, COVID-19

## Abstract

Low-cost mechanical ventilators have been developed in order to deal with the shortage of traditional ventilators whose quantity is not sufficient in an emergency context in Perú. Protofy, a company from Spain, designed one of the first low-cost mechanical ventilation systems OxyGEN which was approved by a medicine agency in its country in special context of COVID 19. Therefore, as main of this article, a redesign of this system named OxygenIP.PE was carried out according to local requirements and available technology, but maintaining its working concept based on compression mechanism by cams. Sensors were added and a control algorithm of the respiratory rate was developed. Ventilation curves monitoring over time was implemented; in this sense, a mathematical model of the whole system was developed. OxygenIP.PE was redesigned, fabricated, and tested measuring its ventilation curves over time. Results indicate that this redesign provides a sturdy equipment able to work during a longer lifetime than the original. The replicability of the ventilation curves behavior is ensured, while the mechanism dimensions are adapted for a particular airbag resuscitator. The mathematical model of the whole system can satisfactorily determine the ventilation curves over time and is used to show the air pressure, volume, and flow as a function of the compression arm's angular position and differential pressure through the breathing circuit measurement, furthermore the algorithms designed as a consequence of the mathematical model were implemented for Raspberry and ARDUINO microcontrollers. There were obtained parameters of pressure 10–65 cmH2O, airflow 50–65 l/m, volume 0–0.5 l, at two values of beat per minute (BPM) 15 and 25.

## 1 Introduction

The first ventilator prototypes were studied many years ago aiming to supply mechanical ventilation for patients [1], furthermore, to solve the “artificial respiration” problem under emergencies such as respiratory insufficiency. Artificial respiration continued to be in development owing to be a big support for medical staff. During the last century, poliomyelitis was a big problem for the world. One of its complications was having trouble breathing, therefore, artificial respiration proved to be a useful solution. However, in parallel to the development of big artificial respiration equipment, practical models were researched and designed. One of these models was known as Pulmotor [1], which supplied oxygen to the patient by periods. Moreover, new concepts for artificial breathing equipment were proposed, such as mixing CO2 with oxygen for patient treatment. Notwithstanding, this particular application was analyzed carefully before developing commercial models owing to avoid the excess of CO2 in the patient.

Although mechanical ventilators have already been used and the knowledge of its system is widespread and explained, they are very expensive for the average household income and its quantity in medical centers is not sufficient either in Peru [2, 3]. During COVID-19 pandemic, low-cost mechanical ventilators have been developed in order to deal with its shortage, they are not able to perform as well as a conventional mechanical ventilator which have been significantly tested in order to obtain a FDA approval; however, they must comply at least with the main ventilation requirements which have been established for emergency use [4]. Part of these low-cost mechanical ventilators have been based on an airbag resuscitator compression mechanism since one of the first functional prototypes was introduced [5]. This mechanism, in spite of its limitations related to a short period use and ventilation parameters control, possess a high degree of buildability and scalability [6] which is a requirement for a developing country where high technological components are not available due to the import shortage. In this context, OxyGEN, developed by Protofy, was one of the ﬁrst low-cost mechanical ventilators based on compression mechanisms to be medically considered as emergency equipment which could be used for short periods of time in order to save lives while traditional ventilators are not available [7, 8]. However, its main components: mechanical power source and airbag resuscitator with their specific performance are not common. In this sense, different ventilation curves would be produced if components with different specifications were used. Furthermore, this equipment isn't able to provide ventilation parameters monitoring which is required by medical staff.

In this research, a re-engineering of the original OxyGEN project is carried out in order to use available components with different specifications because of the non-availability of the originals and obtain the same ventilation curves since these have been already medically tested, and also to meet the local requirements in Perú: a sturdy equipment able to work for a longer period of time, ventilation parameters monitoring and respiration rate control having in mind the possibility of mass production using local technology.

## 2 Materials and methods

### 2.1 Mechanical ventilation system OxygenIP.PE

OxygenIP.PE is an electromechanical equipment which automates the manual compression of an airbag resuscitator in order to provide airflow to a patient in an emergency context. This is an upgraded version of the OxyGEN low-cost mechanical ventilator developed by Protofy [8], which was adapted to the available technology in Peru; however, the original working concept developed by Protofy was maintained due to the satisfactory medical results previously obtained by them in preclinical tests for animals and humans [7].

**Figure 1:**
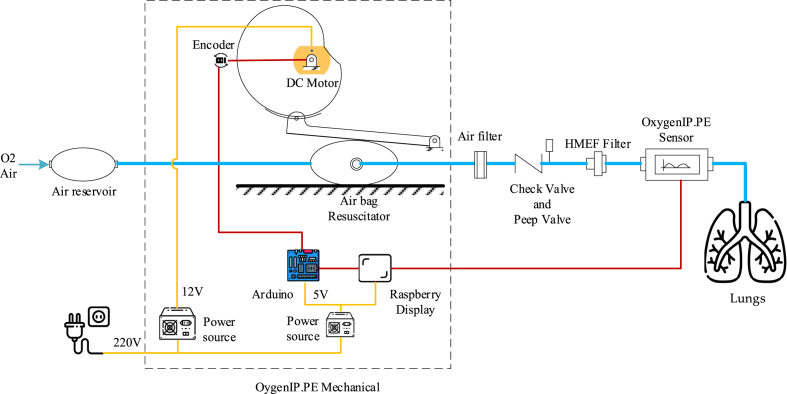
Scheme of the low-cost mechanical ventilation system OxygenIP.PE.

Figure 1 shows a scheme of OxygenIP.PE and its components. This equipment was designed to provide a volume-controlled ventilation (VCV), the main ventilation parameters for the emergency use are available according to [4], these parameters can be selected by the medical staff for the patient requirements.

**Figure 2:**
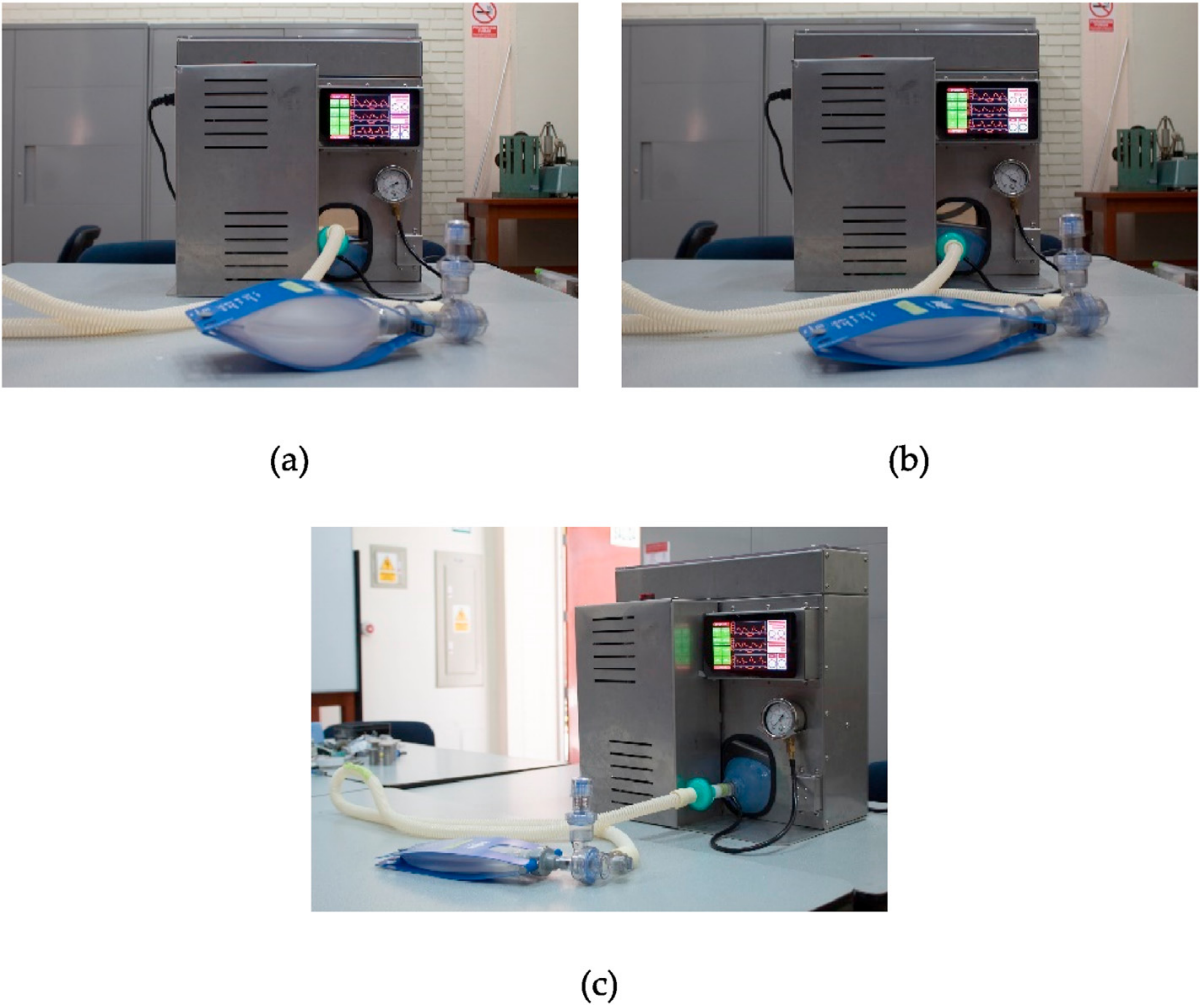
Mechanical ventilation system OxygenIP.PE: (a) Inspiration phase; (b) Exhalation phase; (c) Isometric view.

Figure 2a, Figure 2b, and Figure 2c show frontal and isometric views of the fabricated OxygenIP.PE second prototype during operation, the artificial lung simulates the patient's lung during inspiration state, Figure 2a, and exhalation state, Figure 2b. Its working concept consists of a DC motor which transmits torque and rotation to a cam, this cam drives an oscillating follower, which in turn compresses an airbag in order to increase the air pressure inside and produce an air flow to the patient. The provided ventilation curves to the patient depend on the cam's shape; in this sense, five cams are fabricated following an algorithm developed by Protofy [9]. The cam is changed manually according to the ventilation requirements established by the medical staff and tidal volume, OxygenIP.PE general specifications are shown in Table 1.

**Table 1:** OxygenIP.PE specifications.

Parameter	Specification
Tidal Volume	450–650 mL manually chosen by cam's size
Respiratory rate	Motor speed control 10–30 rpm
Monitoring	Air pressure, volume and flow over time shown in OxygenIP.PE display
Inspiratory Pressure	Up to 40 cm${\text{H}}_{2}\text{O}$
PEEP	0–20 cm${\text{H}}_{2}\text{O}$
$O_{2}\%$	21–100%

#### 2.1.1 Overview

**Figure 3:**
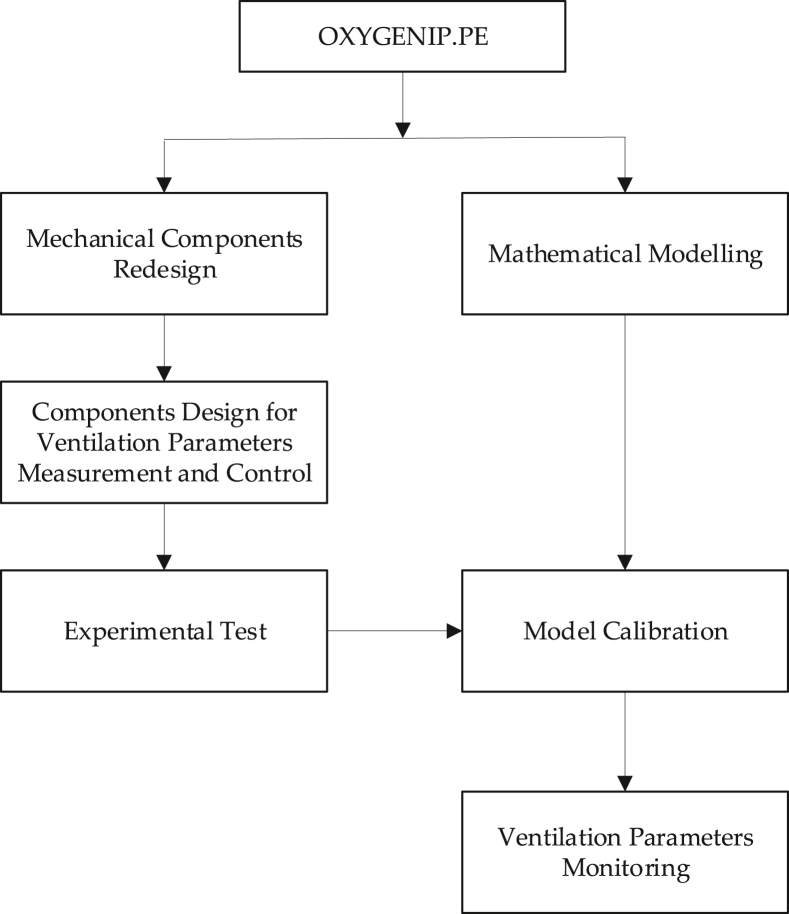
Overview of redesign and upgrade procedure.

An overview of the main phases of the OxygenIP.PE development is shown in Figure 3. The first phase consists of the redesign of the mechanical components in order to produce the same ventilation curves as the original equipment, which had already been validated for emergency, perform a better dynamic behaviour over time and produce a sturdy equipment able to be used for a longer lifetime. At the same time, a mathematical modelling of the mechanical ventilation system based on cams is done. This analysis establishes the relation between physical parameters of the ventilation system and the ventilation parameters which can be produced over time.

In the second phase, electronic components for the measurement of ventilation parameters are added to the system in order to provide useful information to the medical staff; in this sense, a flow meter based on orifice plate was developed and sensors are installed. An angular velocity control of the cam's shaft is also designed and implemented. Then, the ventilation system is tested and the ventilation curves for different lung conditions are obtained. This data is used for the mathematical model calibration where constants are determined. Finally, the mathematical model is used for data treatment of ventilation parameters measurement. Hence precision and accuracy of the ventilation parameters monitoring during operation are ensured.

### 2.2 Components description

#### 2.2.1 Power sources

**Figure 4:**
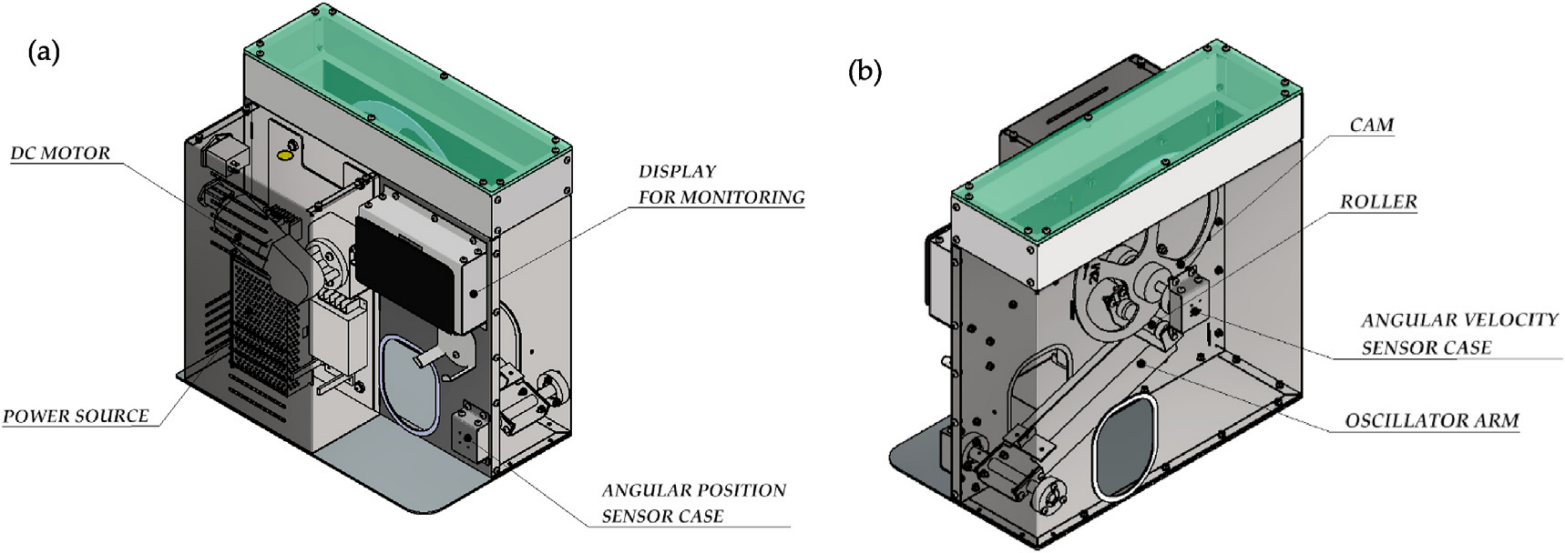
Isometric views of the mechanical ventilation system OxygenIP.PE: (a) View of the frontal components; (b) View of the back components.

Figura 4a shows the isometric view of the OxygenIP.PE where power sources are installed. A 12V@20A power supply is used to energize the motor and sensors. A DC motor Valeo 12V, previously used as a wiper washer motor, is used to provide torque and rotation to the cam compression mechanism, this motor was used instead of DC motor DOGA and an angular velocity control was added. A 5V@5A power supply is used for the Raspberry pi 7″ display.

#### 2.2.2 Airbag compression mechanism

Figure 4b shows the compression mechanism inside the ventilation system. It consists of a cam placed on the DC motor shaft and an oscillating follower with a fixed end to the wall of the ventilator case. An airbag resuscitator BESMED BE-2100 1600mL was used instead of the airbag resuscitator (originally used in the OxyGEN project). BESMED is the most available bag resuscitator in Peru; however, there are some differences between this and the original: volume capacity, dimensions and shape. The mechanism dimensions were originally designed and experimented with a BESMED airbag. In this sense, a different behaviour of the ventilation curves would be produced if the mechanism hadn't been adapted.

The oscillator arm, bearing supports and shafts were redesigned in order to produce a sturdy equipment. The roller is also covered with vulcanized rubber in order to reduce friction, vibrations and noise in operation for a better control and use of the DC motor. From the mechanical design point of view, the machine performance not only depends on how the main function is completed but also in its components' behaviour which are in constant contact during motion and finally influence the precision and accuracy of the ventilation curves over time. Although this is an emergency equipment and must be used in patients for a short period of time, it could be used for a longer lifetime by replacing the airbag. AISI 308S stainless steel was used in order to comply with the medical material requirements.

#### 2.2.3 Respiration rate control

In this mechanism, the respiration rate is governed by the cam's rotation frequency. Since during the airbag compression, the pressure inside it increases, the DC motor performance is affected and its angular velocity leads to decrease. Thus, a control algorithm was developed in order to establish a set rotation frequency and maintain it during operation. AS 5147 encoder was installed to the cam's shaft in order to provide the angular velocity over time. The control algorithm receives the current angular velocity and calculates the correction signal for the DC motor.

#### 2.2.4 Ventilation parameters monitoring

During early March, neither differential pressure sensors, nor absolute pressure sensors (both used to obtain ventilation curves) were available. In this sense, the angular position also allows to predict the ventilations curves using a calibrated mathematical model developed in this research. After a transduction stage, signals from sensors are sent to a display Raspberry 7″ 800 × 480 to show the ventilation curves which bring useful information to the medical staff.

### 2.3 Conceptual model of the compression mechanism

The compression mechanism was designed as a cam-oscillator arm mechanism by Protofy, the cam's shape generation is well developed in its Matlab algorithm [6].

**Figure 5:**
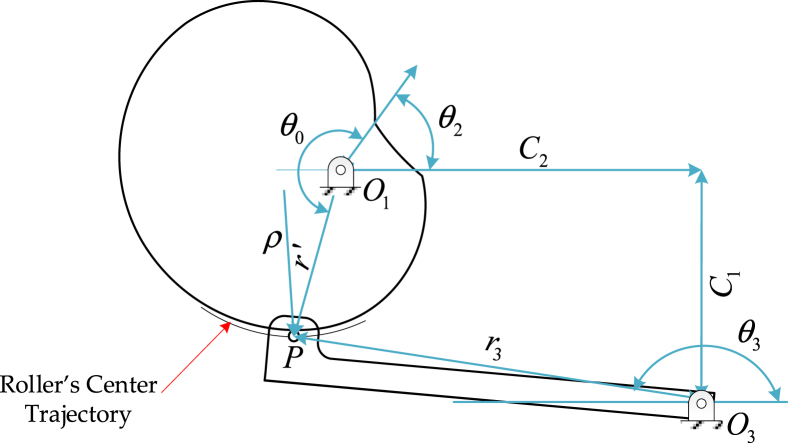
Compression mechanism model.

In order to replicate the original ventilation curves: pressure, volume and flow over time, it is necessary to have knowledge of the dynamic behavior to relate physical variables, such as dimensions, distances and positions, to ventilation curves. Figure 5 shows a conceptual model of the cam-oscillating follower mechanism, where O3 and O1 are the rotation centers of the cam and oscillator arm respectively, C1 and C2 are vertical and horizontal distances between centers, respectively, r’ is the distance between O1 center and roller's center, theta 2 is the cam's angular position, theta0 is the cam's primitive angle, theta p is the angular position to roller's contact point, theta3 is the oscillator arm's angular position r3 is the oscillator arm's length and rho is the curvature radius.

While the follower and cam's shape are in contact, their motions are related, this is achieved by a spring inside the ventilator case. From the model, the following Eqs. (1), (2), and (3) which govern the mechanism kinematics are obtained.

(1) $$r^{\prime }\cos\;\theta_{p}-r_{3}\;\cos\;\theta_{3}=C_{2}$$

(2) $$r^{\prime }sen\theta_{p}-r_{3}sen\theta_{3}=C_{1}$$

(3) $$\theta_{p}=\theta_{2}+\theta_{0}$$

Knowing r’ as a function of theta0, the relation between cam's rotation and arm's rotation is calculated. Using experimental tests, the ventilation curves are obtained as a function of arm's rotation then the dimensions are calibrated in order to produce the same behaviour as original in spite of a different airbag resuscitator or DC motor.

### 2.4 Mathematical modeling of OxygenIP.PE

**Figure 6:**
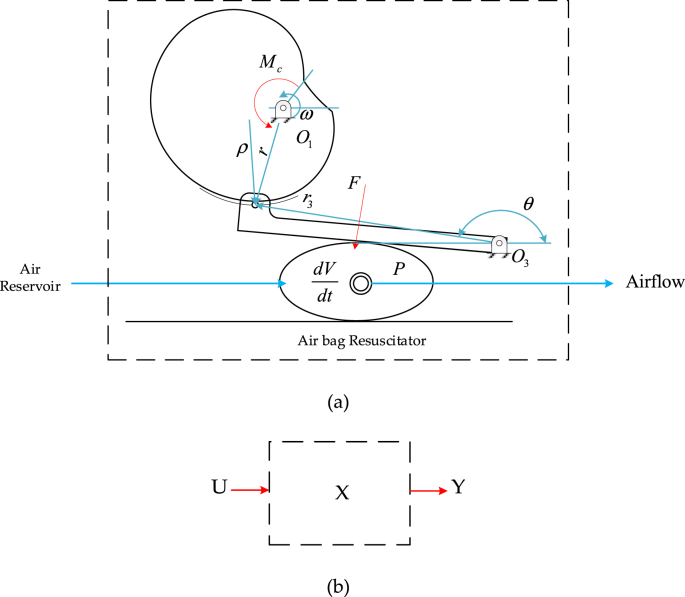
Breathing scheme: (a) Conceptual model of OxygenIP.PE; (b) Input and Output variables of OxygenIP.PE.

Describing the breathing process is a very complex task. To do that, a detailed analysis of the physical variables involved in the transfer of mechanical energy in the artificial mechanism is needed. This way, it is possible to determine the relation between these physical variables from the mechanism and the ventilation parameters, which is finally the aim of the machine. Figure 6a describes the physical variables of the airbag while Figure 6b shows its equivalent as an open loop in order to prepare a mathematical model to organize every variable involved in the mechanical ventilation system. The force “F”, produced by the oscillating follower to the airbag, induces pressure on the airbag (resuscitator), as consequence, the air exits the bag and is goes into the mechanical ventilation system with a pressure “P” and an air volume flow “dV/dt” Figure 6a. The process above is depicted by inlet variables “U” into the system “X” and responses “Y”, Figure 6b.

#### 2.4.1 Procedure description

**Figure 7:**
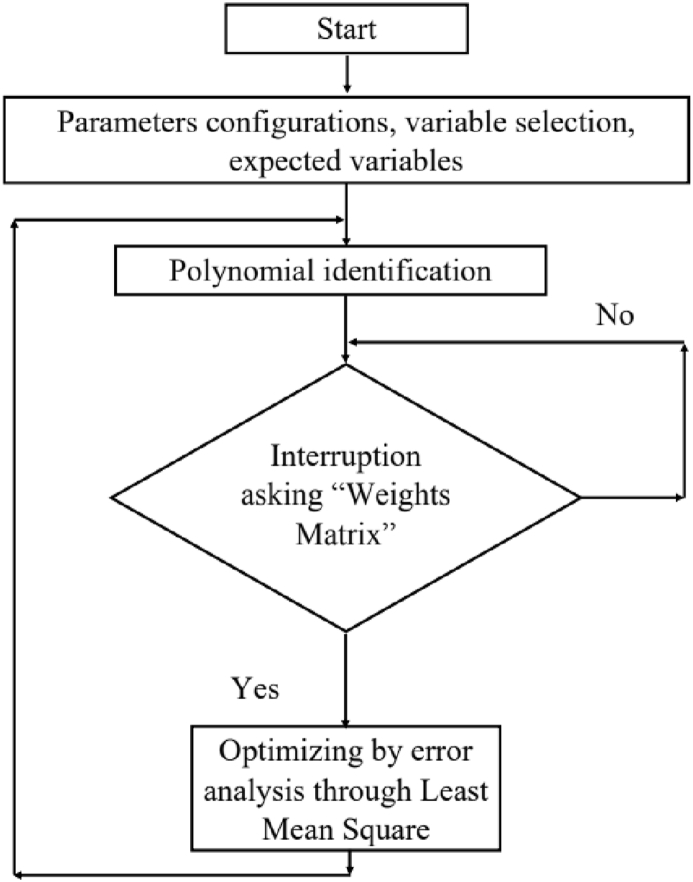
Algorithm for physical parameters estimation for OxygenIP.PE.

The following flowchart, depicted by Figure 7, summarizes the mathematical model designed to obtain an estimate of the parameters and physical variables involved in the mechanical ventilator. The main target of the algorithm is to get the estimate of the mechanical ventilator parameters “air volume, air pressure and air volume flow” relevant for the artificial breathing of patients. For this task, it is necessary to define certain variables and parameters which are conditions for the polynomial model (modulating function proposed in this research). The obtained mathematical model was then calibrated or “corrected” with experimental data averaging different parameters with assigned weights.

#### 2.4.2 Modeling

It is explained in detail from “the mathematical analysis of the system designed to achieve the optimal result of the data monitoring and the ventilation work”, hence, for this reason, it was used advanced methodologies to optimize the process [9, 10] as it is described in the following paragraphs.

The geometrical parameters for a general cam (an element that causes the force over the airbag) was represented in Figure 6a, where ρ is the “curvature radius”, “r” is the rotational displacement, “ω” is the rotational speed [11].

Eq. (4) represents the energy transmission from the cam movement Mc to the energy that needs the volume control Mvc to proportionate oxygen to the patient. However, losses during energy transmission are considered.

(4) $$M_{c}=M_{vc}+losses$$

Eq. (5) is proposed for the energy balance, “f” is the function that depends on the cam rotational speed “ω”, the contact area “s”, and pressure transmission “P”, furthermore the volume flow “dv/dt” and losses γ.

(5) $$f_{w,s,P}+\frac{dv}{dt}=\gamma$$

In this context, Eq. (6) proposes the pressure as a function of “s”, cam mass “M” and curvature radius “ρ”.

(6) P(t)=f(s, ​M, ​ρ)

For which, the curvature radius is described by Eq. (9), and “r” is the rotational displacement [11].

(7) $$\rho =\frac{{\left(r^{2}+{\left(\frac{dr}{dt}\right)}^{2}\right)}^{\frac{3}{2}}}{r^{2}+2{\left(\frac{dr}{dt}\right)}^{2}-r\frac{d^{2}r}{dt^{2}}}$$

By other side, the “diffusion equation” is described by Eq. (8) [12].

(8) $$\frac{\partial P\text{ ​}}{\partial t}=D\frac{\partial^{2}P}{\partial x^{2}}+\mu \frac{\partial P}{\partial x}+\beta \left(P-Pb\text{ ​}\right)=\Gamma$$

And a theoretical solution given by “P(x,t)” is shown by Eq. (9).

(9) $$P\left(x,t\right)\text{ ​}=\frac{4P_{0}}{\pi }\sum_{n=0}^{\infty }\frac{1}{2n+1}e^{-D\frac{\pi^{2}t}{l^{2}}}\left(\frac{\left(2n+1\right)\pi x}{l}\right)$$

And solution of the equation above is the volume flow that is described by Eq. (10).

(10) $$\frac{\partial V}{\partial t}\text{ ​}=\text{ ​}\left(\frac{\delta P}{R}\right)e^{-\frac{t}{\tau }}$$

Therefore, by correlating the last two equations as “Modulating Functions” a general solution can be described: Eq. (11). Furthermore, this equation was widely used in the older dynamical analysis [10, 12, 13].

(11) $$P^{n}y\left(t\right)+\sum_{j=1}^{n}a_{j}P^{n-j}y\left(t\right)=\sum_{j=1}^{n}b_{j}P^{n-j}u\left(t\right)+e\left(t\right)$$

However, as stated by the “Modulating Function solution analysis for diffusion equation” [10, 12, 13], with this methodology, the diffusion equation was solved to get models adapted for specific applications. By obtaining the solutions of the coefficients, it can be possible to get information of the geometrical, thermal and mechanical parameters which can be adjusted to different necessities and applications in fluid mechanics. That is the reason why the Modulating Function in this analysis gives plenty of strategies. The Eq. (12) describes “space fractional advection dispersion equation” c.

(12) $$\frac{\partial c\left(x,\text{ ​}t\right)}{\partial dt}+v\left(x\right)\frac{\partial c\left(x\right)}{\partial dx}-d\left(x\right)\frac{\partial c{\left(x\right)}^{\alpha }}{\partial dx^{\alpha }}=r\left(x,\text{ ​}t\right)$$

Therefore, proposing Eq. (13) to find solutions for the mechanical fluid variables [10, 12, 13].

(13) $$\frac{\partial^{\alpha }}{\partial x^{\alpha }}f\left(x\right)\text{ ​}=\frac{1}{\Gamma \left(n\text{ ​}-\text{ ​}\alpha \right)}\frac{d^{n}}{dx^{n}}\text{ ​}\int_{x=0}^{x}{\left(x\text{ ​}-\text{ ​}t\right)}^{n-\alpha -1}f\left(t\right)dt$$

in which, having “g” as the “Modulating function”, it can be possible to further reduce equations such as described in Eq. (14).

(14) $$\int_{x=0}^{I}g\left(I-x\right)\frac{\partial^{\alpha }}{\partial x^{\alpha }}f\left(x\right)=\int_{x=0}^{I}\frac{\partial^{\partial^{\alpha }}}{\partial x^{\alpha }}f\left(I-x\right)dx$$

Furthermore, a reduced differential equation is given in Eq. (15)

(15) $$\frac{\partial^{\alpha }}{\partial x}\frac{\partial^{\partial^{\alpha }}f\left(x\right)}{\partial x^{\alpha }}=\psi_{o}\left(n\text{ ​}-\text{ ​}\alpha \right)\frac{\partial^{\alpha }}{\partial x}\frac{\partial^{\alpha }f\left(x\right)}{\partial x^{\alpha }}-G$$

In which, “G” is given by the Eq. (16)

(16) $$G=\frac{1}{\Gamma \left(n\text{ ​}-\text{ ​}\alpha \right)}\frac{d^{n}}{dx^{n}}\text{ ​}\int_{x=0}^{x}{\left(x\text{ ​}-\text{ ​}t\right)}^{n-\alpha -1}\;\ln\left(x-t\right)f\left(t\right)dt$$

Finally, to solve the diffusion equation, the coefficients of the general diffusion equation are given by Eq. (17)

(17) $$\nu \left(x\right)=\sum_{k=1}^{k_{o}}v_{k}f_{k}\left(x\right)$$

And Eq. (18)

(18) $$d\left(x\right)=\sum_{k=1}^{k_{o}}pkf_{k}\left(x\right)$$

Therefore, by linear algebra (because of the Modulating Functions solutions) in Eq. (19)

(19) QX=Y

In which “Q” is given by Eq. (20)

(20) $$Q=\left(\begin{array}{lllll} q_{11} & q_{12} & q_{13} & ... & q_{1k_{1}+k_{2}}\\ q_{21} & q_{22} & q_{23} & ... & q_{2k_{1}+k_{2}}\\ \vdots & \vdots & \vdots & ... & \vdots \\ \vdots & \vdots & \vdots & \vdots & \vdots \\ q_{\cdots 1} & q_{\cdots 2} & q_{\cdots 3} & ... & q_{\cdots k_{1}+k_{2}} \end{array}\right)$$

Also “X” is described by Eq. (21)

(21) $$X=\left(\begin{array}{l} v_{1}\\ v_{2}\\ v_{3}\\ \vdots \\ v_{k1}\\ d_{1}\\ d_{2}\\ d_{3}\\ \vdots \\ d_{k2} \end{array}\right)$$

Furthermore “Y” is given by Eq. (22)

(22) $$Y=\left(\begin{array}{l} y_{1}\\ y_{2}\\ y_{3}\\ \vdots \\ y_{\cdots } \end{array}\right)$$

The Eq. (23) is proposed to obtain every component “q” from Eq. (20)

(23) $$q_{m,k}=\left\{ \begin{array}{l} \int_{x=0}^{L_{1}}\frac{\partial fk\left(x\right)\varphi_{m}\left(L_{1}-x\right)}{\partial x}c\left(x,t\right)dx\\ \int_{x=0}^{L_{1}}\frac{\partial^{\alpha }\left(x\right)\varphi_{m}\left(x\right)}{\partial x}c\left(L_{1}-x,t\right)dx \end{array}\right.\begin{array}{l} ,\;k=1,2,\;...,\;k_{1}\\ ,\;k=k_{1}+1,\;...,\;k_{1}+k_{2} \end{array}$$

Moreover, the solution “y” is given by Eq. (24)

(24) $$y_{m}=-\int_{x=0}^{L_{1}}\varphi_{m}\left(L_{1}-x\right)\left(r\left(x,t\right)\frac{\partial c\left(x,t\right)}{\partial t}\right)dx$$

In which φ is described by Eq. (25)

(25) $$\varphi_{m,k}\left(L_{1}-x\right)=p_{k}\left(x\right)\varphi_{m}\left(L_{1}-x\right)$$

Therefore, the optimal parameters “X” are obtained through Eq. (26)

(26) $$X={\left(Q^{T}Q\right)}^{-1}Q^{T}Y$$

Nevertheless, to adapt the estimates of desired parameters it is necessary to analyze the error between the desired answer and the expected or measured values. Eq. (27), where solution error analysis “e(t)” is the discrete error, and “V” keeps the Fourier series coefficients [10, 12, 13], tasks this issue.

(27) $$e_{n}\left(m\right)=\sum_{k=m}^{n+m}\alpha \left(k,m,\theta_{a}\right)V\left(k\right)$$

Furthermore, α is the frequency parameter function that is described by Eq. (28)

(28) $$\alpha \left(k,m,\theta_{a}\right)=C_{k}-m{\sum_{j=0}^{n}a_{j}\left(jkw_{o}\right)}^{n=j}$$

For which, the nonlinear model for error analysis is given in Eq. (29)

(29) $$\sum_{j=0}^{n_{1}}\sum_{k=1}^{n_{2}}g_{j\left(\theta \right)}F_{jk}\left(u,y\right)P_{jk}\left(p\right)E_{k}\left(u,y\right)=0$$

Hence, the cost function described through Eq. (30) given to enhance the parameters θ [10, 12, 13].

(30) $$J\left(\theta \right)=\sum_{j=0}^{n_{1}}\sum_{k=0}^{n_{2}}r_{jk}g_{j}\left(\theta \right)g_{k}\left(\theta \right)$$

Also, according to get “parameters of the main model” the derivation is described by Eq. (31)

(31) $$\frac{\partial J}{\partial \theta }={\left(Y-\Gamma \theta \right)}^{T}W^{-1}\left(Y-\Gamma \theta \right)$$

Where parameters are shown in Eq. (32), as dependent of the “adaptive” coefficients

(32) $$\theta ={\left(\Gamma^{T}W^{-1}\Gamma \right)}^{-1}\Gamma^{T}W^{-1}Y$$

For which, the optimal response is given by Eq. (33)

(33) $$\overset{\wedge }{Y}=X{\left(X^{T}X\right)}^{-1}X^{T}ϒ$$

And the adaptive optimal solution (to achieve the parameters) is obtained through Eq. (34). For this reason, all the parameters which are looking for the estimated answer of the mechanical ventilator through the adaptive matrix weight “W” [10, 12, 13].

(34) $$\overset{\wedge }{Y}=X{\left(X^{T}W^{-1}X\right)}^{-1}X^{T}W^{-1}ϒ$$

### 2.5 Ventilation curves estimation based on mathematical model

**Figure 8:**
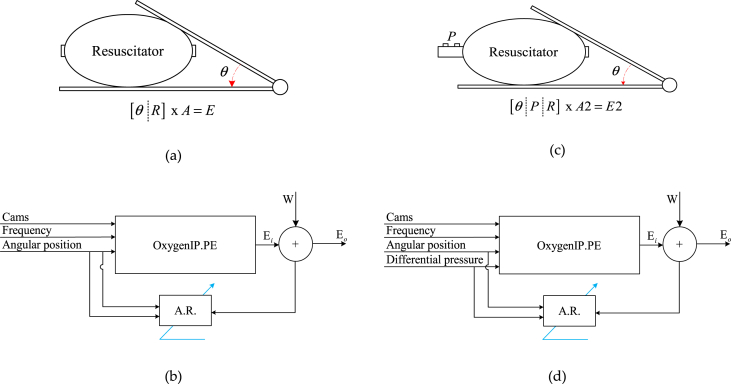
(a) Scheme for estimations with angular position as main input variable; (b) Block diagram for estimations with angular position as main input variable to design sample variables algorithm; (c) Scheme for estimations with angular position and differential pressure as main input variables; (d) Block diagram for estimations with angular position and differential pressure as main input variables to design sample variables algorithm.

It was evaluated the performance of the mathematical model (after to design the model more appropriate for this mechanical system) through the design of an algorithm to estimate: “air volume, air pressure and airflow” furthermore the estimated parameters were evaluated with the identified parameters by experiments according to enhance the answer of the system. In Figure 8a is depicted the scheme of the linear equation (achieved as a consequence of the model and algorithm designed) which let to estimate the behavior of the physical variables (volume, pressure and airflow). “θ” is the angular displacement of the rotor over the airbag, “R” is the reference matrix which contains the desired (theoretical) values of the physical variables, “A” has the matrix weights to get adaptation and “E” is the matrix which contains the final estimation.

According to find better estimation of the physical variables: “volume, pressure and airflow”, it was prepared the mathematical model as dependence of two measurements: “the rotor angle displacement and the differential pressure” that are included in the entrance matrix as reference of the estimation by adaptive weight compensations. That is the reason why in Figure 8c is depicted the scheme of the entrance variable matrix composed by θ, “P” and “R” and the adaptive matrix “A” and the result of estimations given by “E”.

Moreover, Figure 8d shows the algorithm by graphic representation, in which there are as entrance variables “differential pressure, angular position furthermore the parameter Cams (this is the cam size which is the data setting by the medical doctor) and the frequency (this is the respiration rate which is a chosen by medical staff)”. All these entrance variables and parameters are introduced to the algorithm (designed as a consequence of the mathematical model designed and described in chapters above) the correlation from introduced data with the adaptation matrix “A” and reference matrix “R” achieved the responses matrix “E” which was under correlation with disturbance “W” and the final response matrix “E0”.

**Figure 9:**
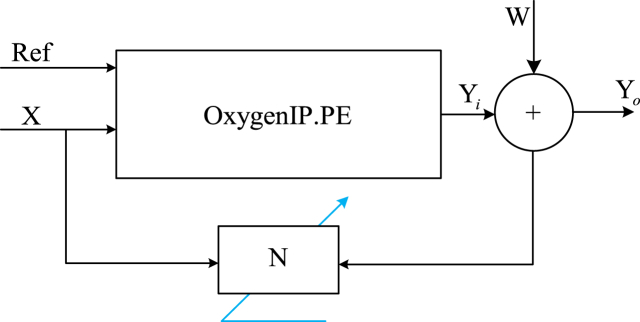
The final multivariable estimator.

Furthermore; the reference signal as input matrix component achieves better estimation in the designed algorithm to measure the physical variables: pressure, flow and volume through the differential pressure analysis. Therefore, in Figure 9 is depicted the multivariable estimator that includes the reference signal “ref”, which must to be part of the input matrix “X” of the mechanical ventilator system. Furthermore, the response “y” added to the disturbance “w” and as the consequence it was achieving the final response system “Yo”.

As a consequence, the control system (which is only for motor speed control) gave the possibility to change the speed of air proportion to the patient. However, it cannot be controlled the time that patients need to achieve air by inharmonic periodicity, therefore it could cause a big delay trouble.

For this reason, the mathematical model of the ventilation expects time delay as the part of disturbance in frequency analysis, it means the “optimal, adaptative and predictive multivariable algorithm enhances the response time of the system in order to provide enough operating time for the main system to avoid also disturbances in time domain”.

By other side, the “adaptive Least Mean Square error analysis helped reduce measurement errors because of an efficient predictive tracker of a reference variables of the ventilation sensors data that was obtained by the main algorithm of the system”. Therefore, the estimation tends to be similar the reference signal due to the reference signal is a part of the input matrix.

## 3 Experimental setup

**Figure 10:**
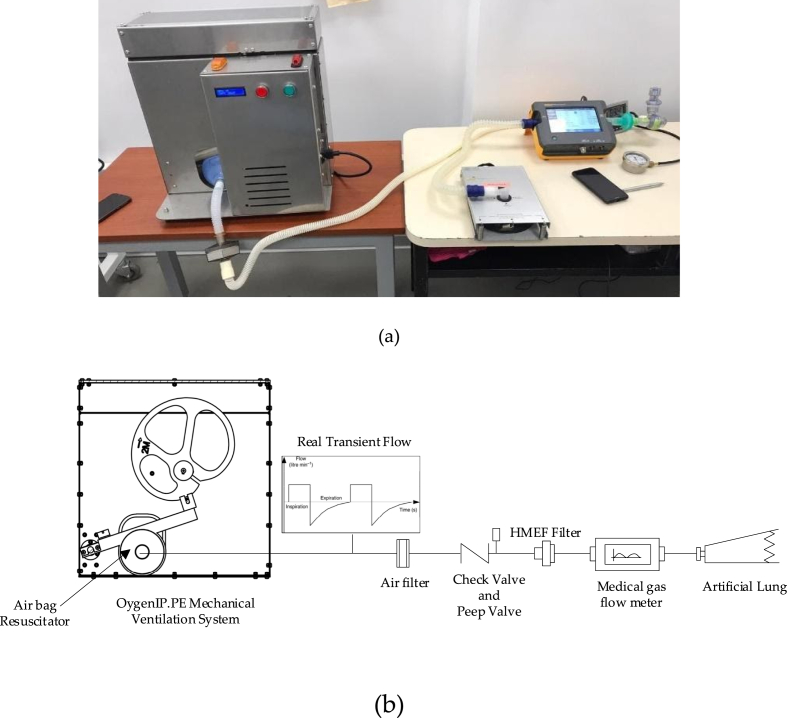
(a) Experimental setup of ventilation curves measurement at the Biomedical Laboratory from the Pontificia Universidad Católica del Perú; (b) Scheme of the experimental setup.

The mechanical ventilation system OxygenIP.PE was tested in the Biomedical Engineering Laboratory from the Pontificia Universidad Católica del Perú. Figure 10a shows the experimental setup and Figure 10b shows an equivalent scheme of the setup and its components. It consists of the system OxygenIP.PE connected to a medical breathing circuit where a medical gas flow meter Fluke VT650 was installed.

An artificial lung Fluke Accu Lung, a precision test lung, was used, it can simulate three conditions: compliance 50 mL/cmH2O and resistance 5cmH2O-s/L, compliance 20 mL/cmH2O and resistance 20cmH2O-s/L and compliance 10 mL/cmH2O and resistance 50cmH2O-s/L. For each condition, all the cam sizes were tested during 2 min per cam size and the ventilation curves air volume, pressure and flow over time were obtained.

## 4 Results and discussion

### 4.1 OxygenIP.PE experimental results

**Figure 11:**
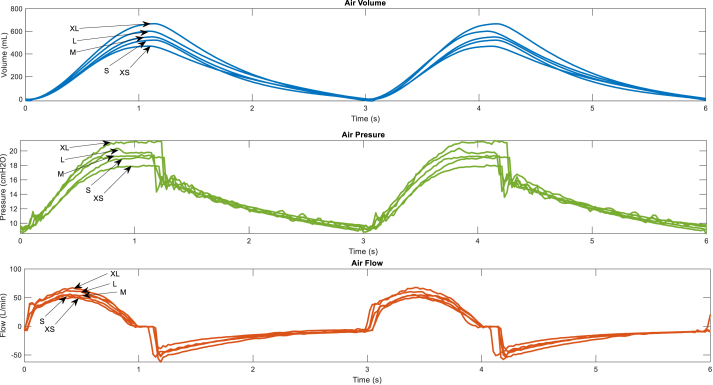
Air volume, pressure and flow curves over time for a lung with compliance 50 mL/cmH2O and resistance 5cmH2O-s/L.

**Figure 12:**
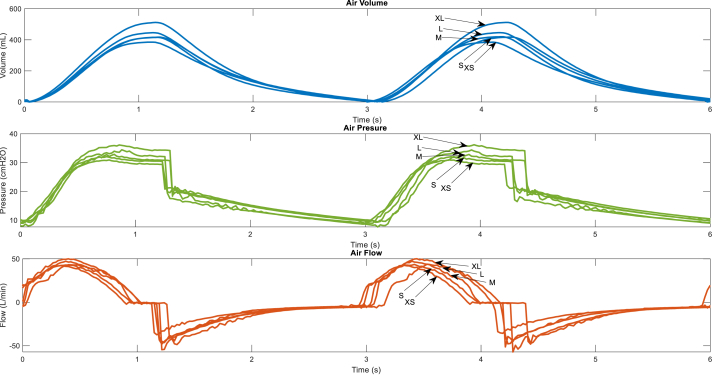
Air volume, pressure and flow curves over time for a lung with compliance 20 mL/cmH2O and resistance 20cmH2O-s/L.

**Figure 13:**
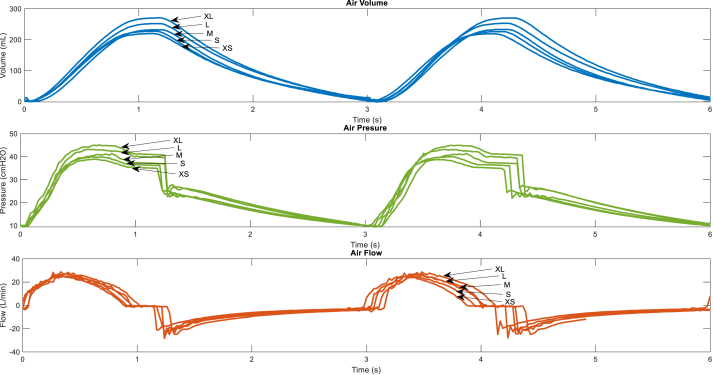
Air volume, pressure and flow curves over time for a lung with compliance 10 mL/cmH2O and resistance 50cmH2O-s/L.

The mechanical ventilation system OxygenIP.PE was tested for 2 min for each cam. A medical flow meter was used for measuring ventilation curves and the results are shown in Figure 11, Figure 12 and Figure 13 for the different characteristics of the artificial lung. Figure 11 shows the ventilation curves for the five cams in a breathing circuit with an implemented artificial lung of a compliance 50 mL/cmH2O and resistance of 5cmH2O-s/L. The minimum and maximum air volume over time are obtained by using the minimum cam size XS and maximum cam size XL, respectively; the same behavior occurs with the pressure and flow over time. Also, the relation between the ventilation variables: volume, pressure and flow are manifested according to the cam size, a higher cam size produces a higher value of the ventilation curves for the same lung characteristics. This is because a higher cam size produces a higher compression of the airbag resuscitator, hence a higher pressure value is produced in the breathing circuit. It is known that compliance and resistance of the lung produces a resistance pressure, also the difference between the airbag pressure and the resistance pressure produces the airflow to the patient; in this sense, ventilation curves increase in value over time.

Figure 12 and Figure 13 show the ventilation curves for a patient with a lung in two conditions; a partially damaged lung with a compliance 20 mL/cmH2O and resistance 20cmH2O-s/L, and a completely damaged lung with a compliance 10 mL/cmH2O and resistance 50cmH2O-s/L. For each lung condition, it can be noted that the shape and relation of the ventilation curves maintain the same behavior as explained before and ventilation values volume, pressure and flow continues increasing while the cam size increases. However, a damaged condition of the lung produces the minimum ventilation values of air volume and flow over time. This is due to the compliance and resistance of the lung which increases the resistance pressure if the lung damage increases, hence the pressure inside the airbag also has to increase in order to provide air flow to the patient; this is why the maximum pressure is obtained for the damaged lung condition.

**Figure 14:**
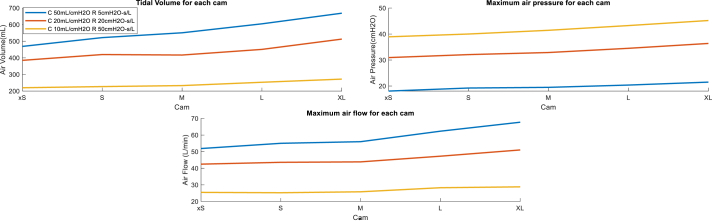
Characteristic ventilation curves for each cam of the OxygenIP.PE mechanical ventilation system.

It's necessary to represent the characteristics of the ventilation curves for the different possible conditions, either for different cams sizes or lung conditions to help medical staff who select the proper cam size for a particular patient. In this sense, Figure 14 shows the highest values of the ventilation curves for each cam size and lung condition. For the same lung condition, air volume, pressure and flow increase while the cam size increase as explained before. However, these values decrease significantly for a damaged lung condition; for the minimum cam size the air tidal volume decreases to 46.91% when the lung is damaged (compliance 10 mL/cmH2O and resistance 50cmH2O-s/L), while for the maximum cam size XL, the air volume decreases to 40.65% its value.

#### 4.1.1 Compression mechanism and air volume behaviour

**Figure 15:**
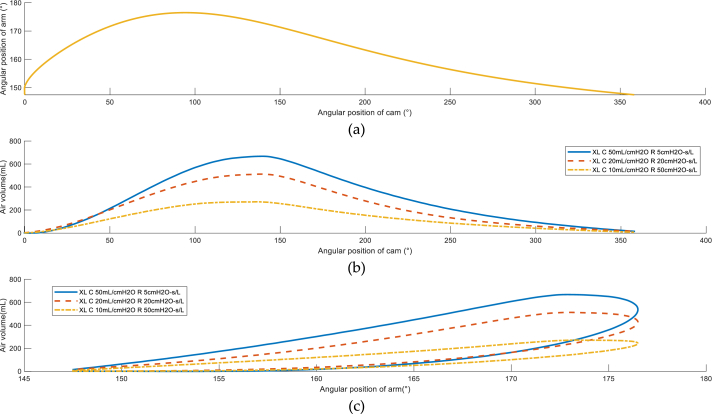
Tidal volume as a function of the oscillator arm angular position.

The motion equations of the mechanism obtained by the conceptual model establish the relations shown in Figure 15a, in Figure 15b and Figure 15c. The oscillator arm's rotation as a function of the cam's rotation angle is shown in Figure 15a. The cam's movement is governed by the DC motor and the angular velocity control which sets an approximately constant value allowing a proportional relation between the cam's angular position and the volume over time produced by the airbag compression, Figure 15b. Hence the air volume can be also plotted as a function of the oscillator arm's rotation angle, Figure 15c. The airbag compression state takes place from 0-150° of the oscillator arm's angle, which is the inspiration phase; then a check valve at the breathing circuit is closed and the exhaust air flow is not able to go back to the airbag. In this sense, since 150°, OxygenIP.PE is not able to control the air flow, but it is measured.

In Figure 15c, up to 175°, linear regression can be considered between the provided air volume through a breathing circuit and the oscillator arm's rotation angle when the ventilator is connected to an artificial lung with these parameters.

### 4.2 Mathematical model

**Figure 16:**
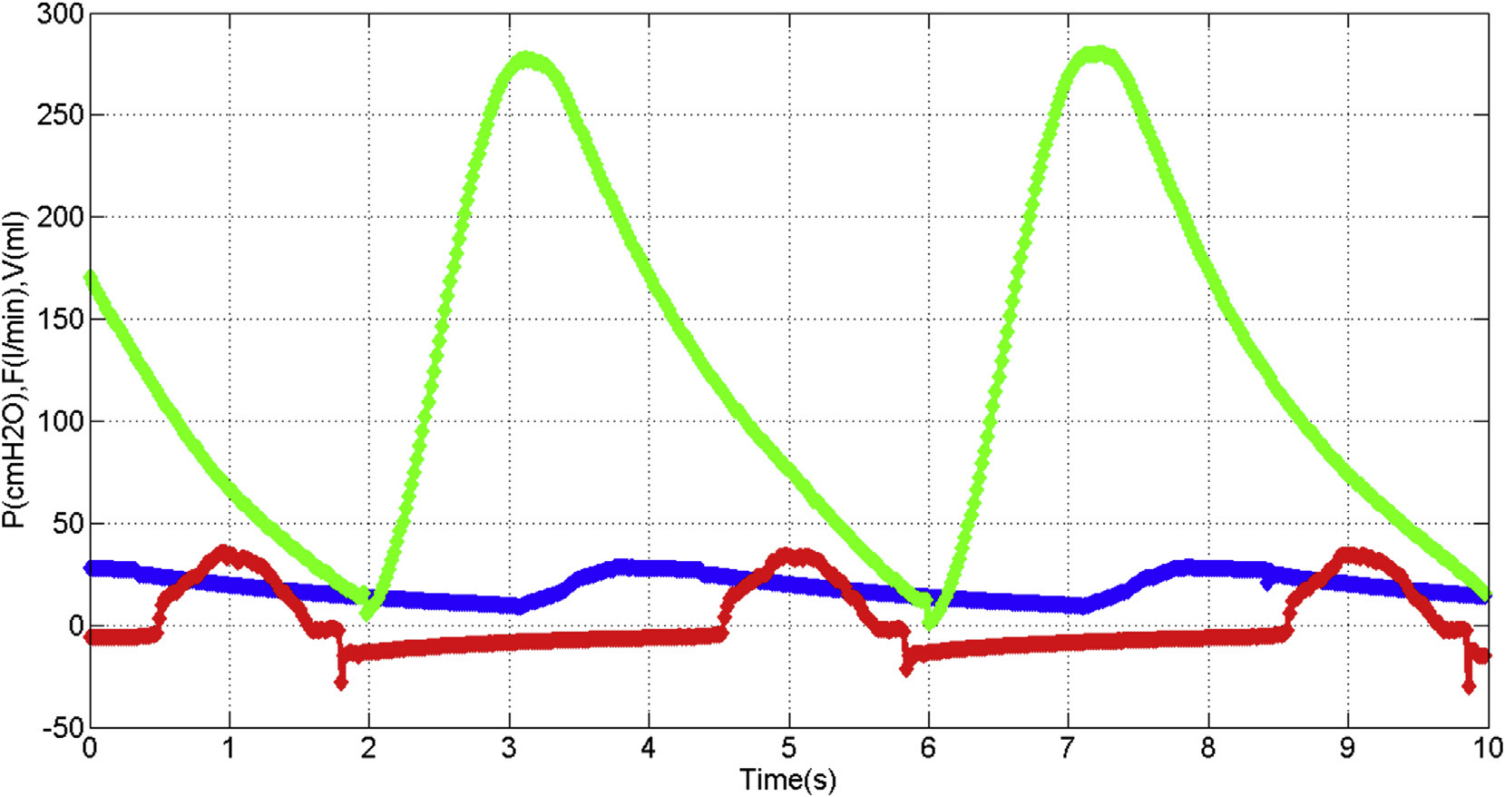
Pressure, volume and flow curves over time by the mathematical analysis above.

Figure 16 shows the pressure (blue color curve) in cmH2O, the air volume flow (red color curve) and the volume (green color curve) which were obtained by the mathematical analysis described by equations that were explained in the paragraphs above. The pressure, volume and airflow were achieved offline by data from FLUKE equipment (due to its internal sensors).

This is the reason, why there are out of phases between them. Nevertheless, in this part of the analysis it was made the identification of the achieved data. In following chapters it is corrected the out of phase due to the adaptive/predictive designed algorithm to make this corrections online and to show physical variables to the medical doctor. The standard error is 0.03 for the airflow, 0.02 for the volume and 0.04 for the pressure.

**Figure 17:**
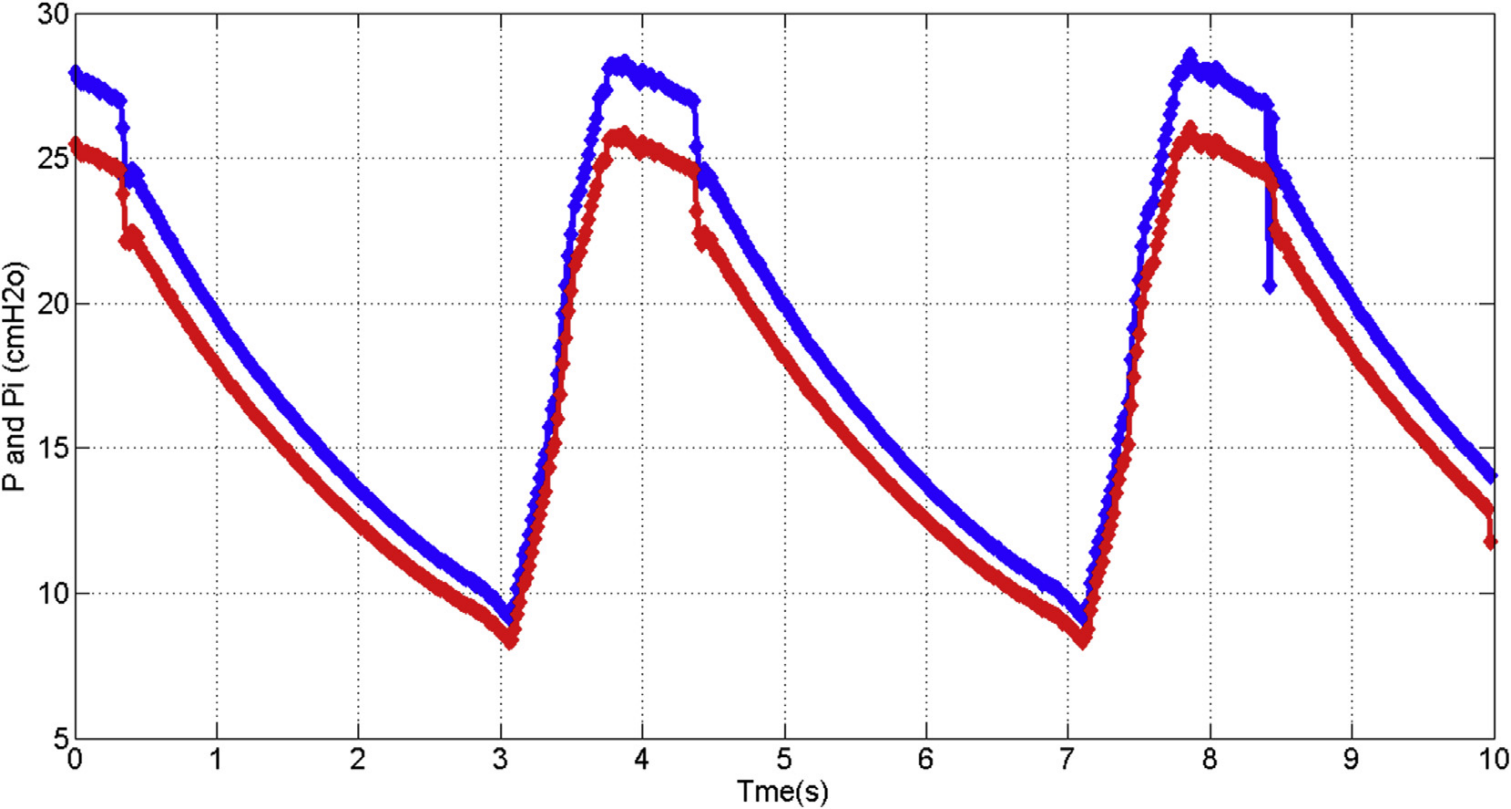
Pressure and identified pressure curves over time by mathematical analysis above.

Furthermore, Figure 17 shows the pressure (blue color curve) and the identified pressure (red color curve) in cmH2O during time of 10 s for measurements and simulation for the identification model. During the identification analysis, it is usual to identify disturbances in the system (that stochastic variables can be corrected after to identify the mathematical model), which correlated by theoretical analysis with experimental data, such as, for example, in the Figure 8 for the intervals 0–1 s, 4–5 s, and 8–9 s there is a sudden pressure drop that can be caused by mechanical or electromechanical disturbances. This information helped to make a feedback in the design and for the main algorithm, because it could get understanding, when it comes an external disturbance (the algorithm gives an alarm for medical doctor) or when it comes a disturbance that is not a part of the breathing monitoring. Therefore, it needs to be corrected by the main monitoring algorithm as it is explained in next chapters. The standard error 0.087 for the pressure.

**Figure 18:**
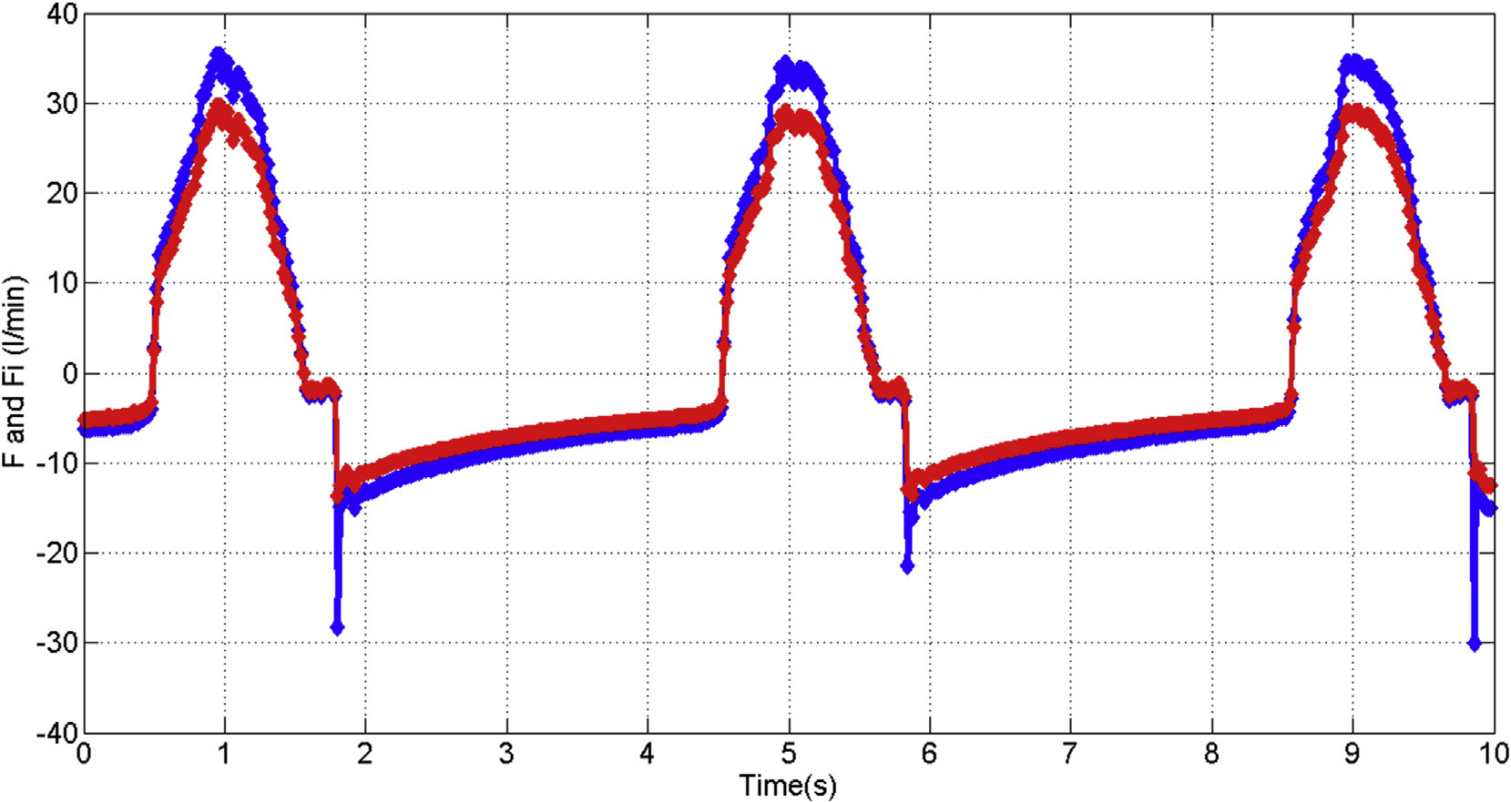
Flow and identified flow curves over time by mathematical analysis above.

Figure 18 shows the air volume flow (blue color curve) and its identification (red color curve) in l/min during 10 s, it is possible to see that the identified airflow curve can avoid possible disturbances (such as rejecting periodical peaks from the blue color curve). As it was explained by the figure above, the identification tasks needs every possible mistake that is necessary to design the mathematical model according to give the report of mistakes in the breathing process, that after it is prevented as disturbances analysis by adaptive/predictive criterion by the main algorithm. As for example, in Figure 18 there is an offset of flow that is not equal to zero, which as the part of the first recognition of the system according to enhance the design and the mathematical model as it was achieved and described in next chapters. The standard error is 0.063 for the airflow.

**Figure 19:**
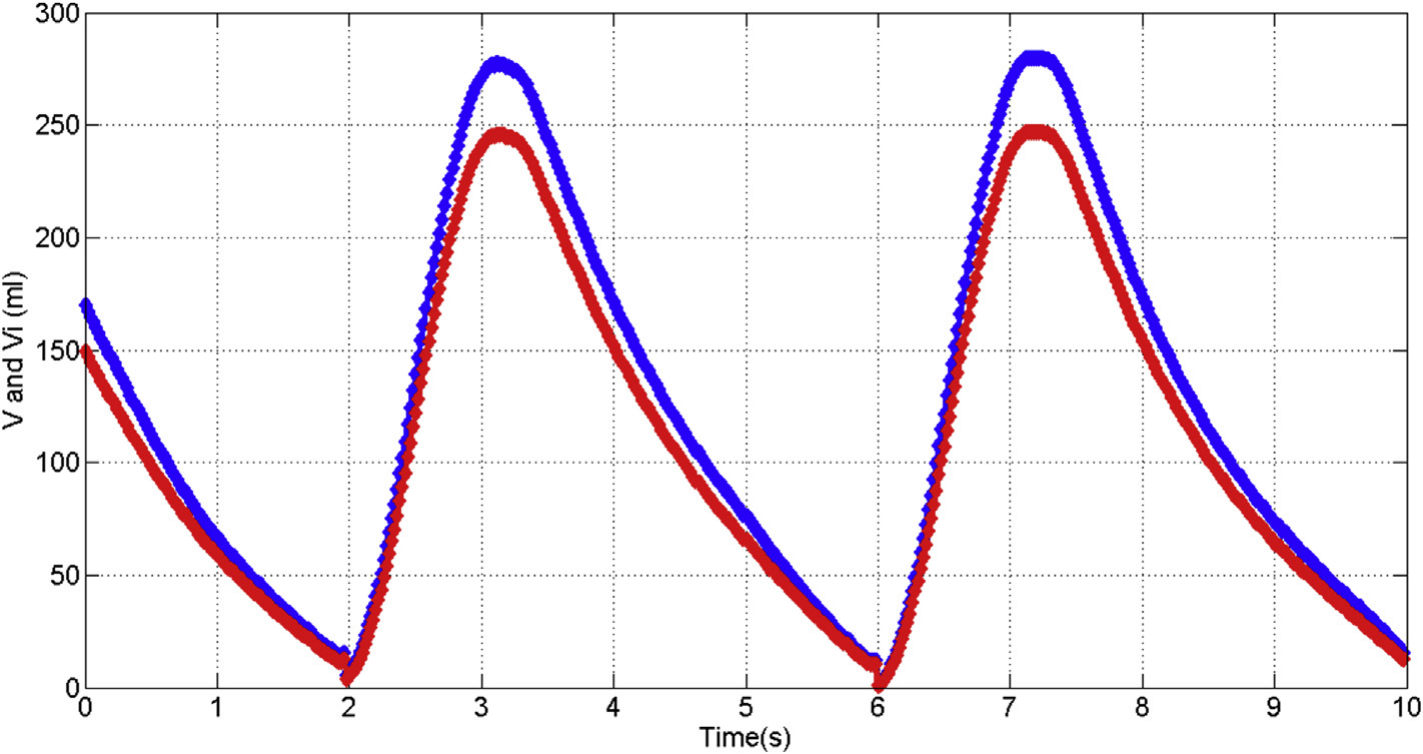
Volume and identified volume curves over time by mathematical analysis above.

Finally, in Figure 19 is shown the volume (blue color curve) and its identification (red color curve) in (mL) during time of 10 s for measurements and simulation for the identification model. Moreover, as it was analyzed for the last two identified variables, the volume also was identified with every disturbance around the system, because of to enhance after the recognition of every perturbation, which gives information for the main algorithm to give an alarm for the medical doctor or to make a filter, while it is recognized disturbance as the electromagnetic noise, for example. Therefore, the parametric and not parametric identification helped to achieve the mathematical model according to design the predictive/adaptive algorithm that was used to achieve the data that is described in next chapters. The standard error is 0.023 for the volume.

**Figure 20:**
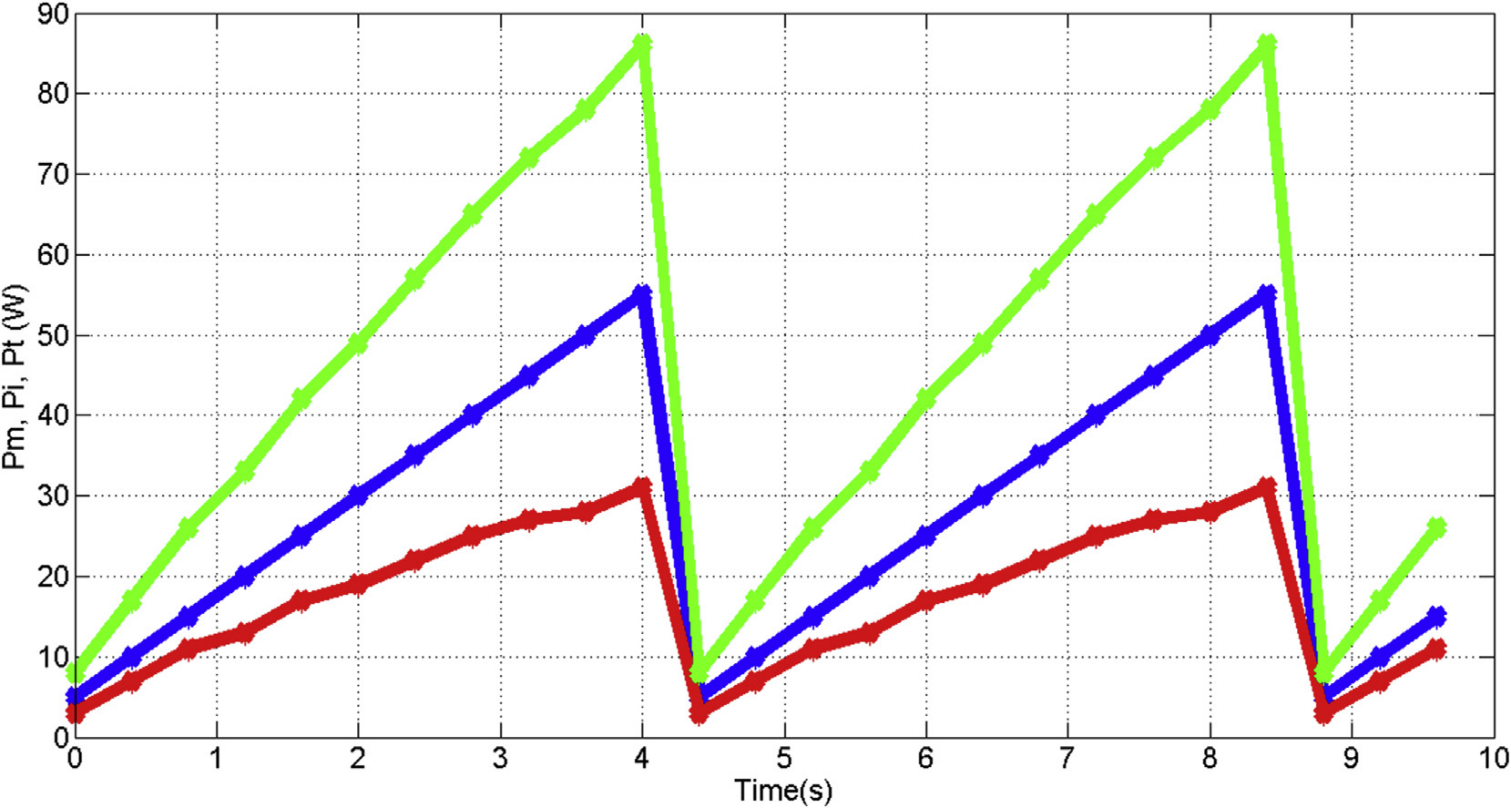
Power analysis of the mechanical ventilator.

By another side, the parametric and non-parametric identification analysis, that was made in this research by the adaptive and polynomial analysis from equations in the paragraphs above [10, 12, 13], this study let to achieve the performance of the mechanical ventilator through the power consumed and described in Figure 20. In which, the blue color curve is the motor power (W), the red color curve is the inertia power (W) and the green color curve is the total power which is less than the maximal power produced by the motor in maximal load.

**Figure 21:**
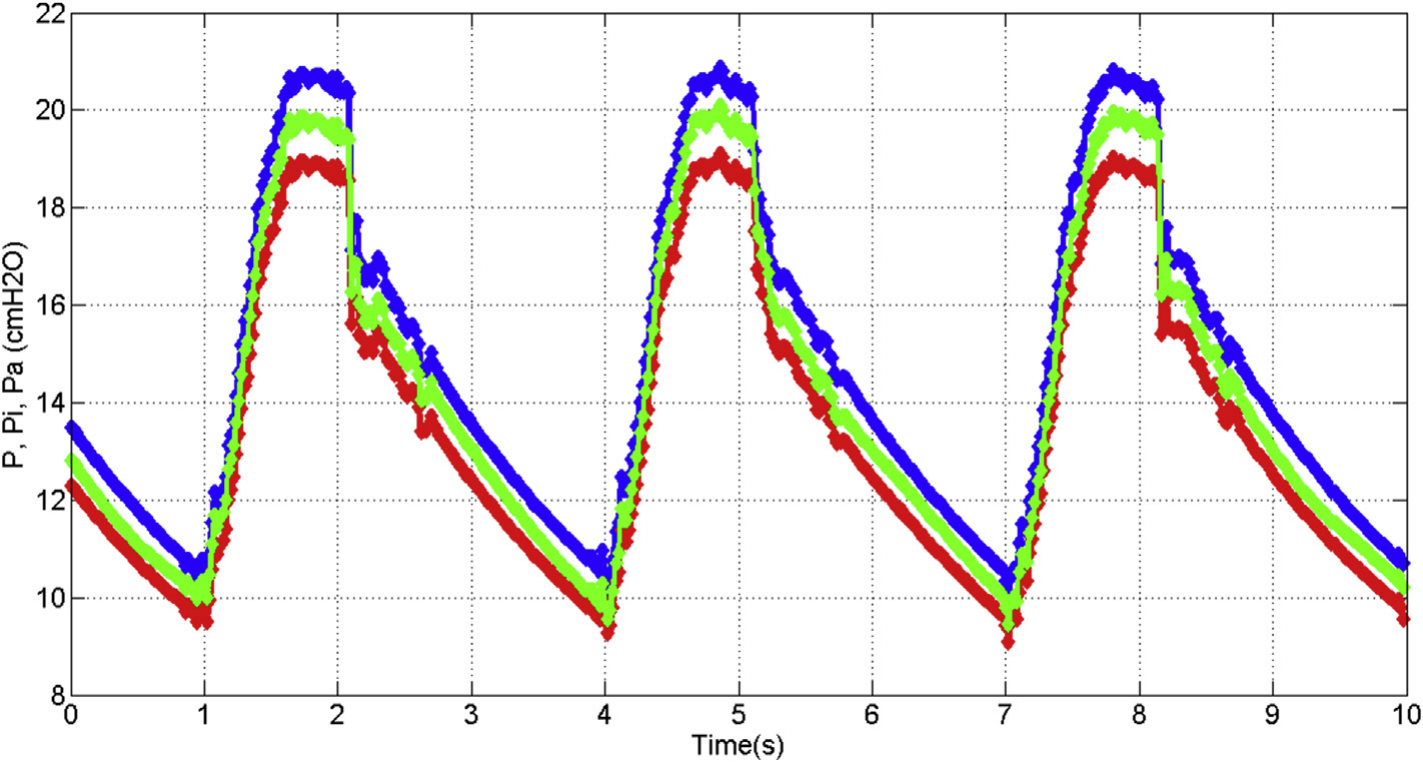
Measured pressure, identified pressure and adaptive-predictive pressure.

Moreover, to enhance the identified answer of the system (for every variable: pressure, airflow and volume) the prediction analysis through adaptive coefficients in the polynomial model was developed. Hence, in Figure 21 is shown the measured pressure (blue color curve), the identified pressure (red color curve) and the adaptive-predictive pressure (green color curve) in cmH2O, during time of 10 s for measurements and simulation for the identification and the adaptive/predictive model. The standard error 0.094 for the pressure.

**Figure 22:**
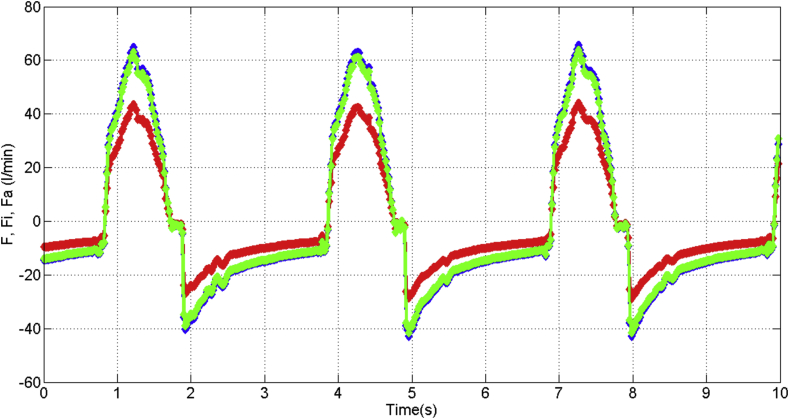
Measured air flow, identified air flow and adaptive-predictive air flow.

In Figure 22 is shown the measured airflow (blue color curve), the identified airflow (red color curve) and the adaptive-predictive airflow (green color curve) in litters per minute, during time of 10 during time of 10 s for measurements and simulation for the identification and the adaptive/predictive model. The standard error is 0.03 for the airflow.

**Figure 23:**
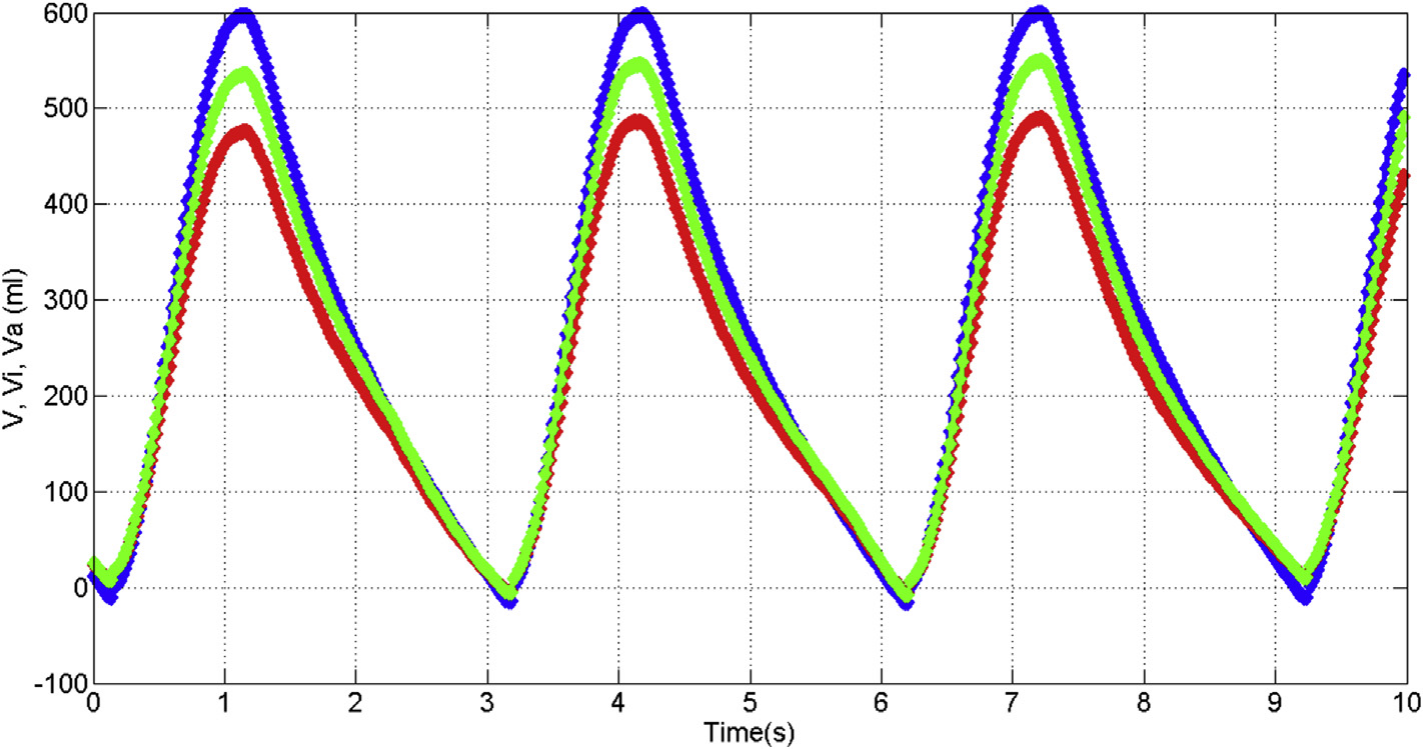
Measured volume, identified volume and adaptive-predictive volume.

In Figure 23 is shown the measured volume (blue color curve), the identified volume (red color curve) and the adaptive-predictive volume (green color curve) in ml, during time of 10 s for measurements and simulation for the identification and the adaptive/predictive model. The standard error is 0.053 for the volume.

### 4.3 Ventilation curves prediction based on the mathematical model

**Figure 24:**
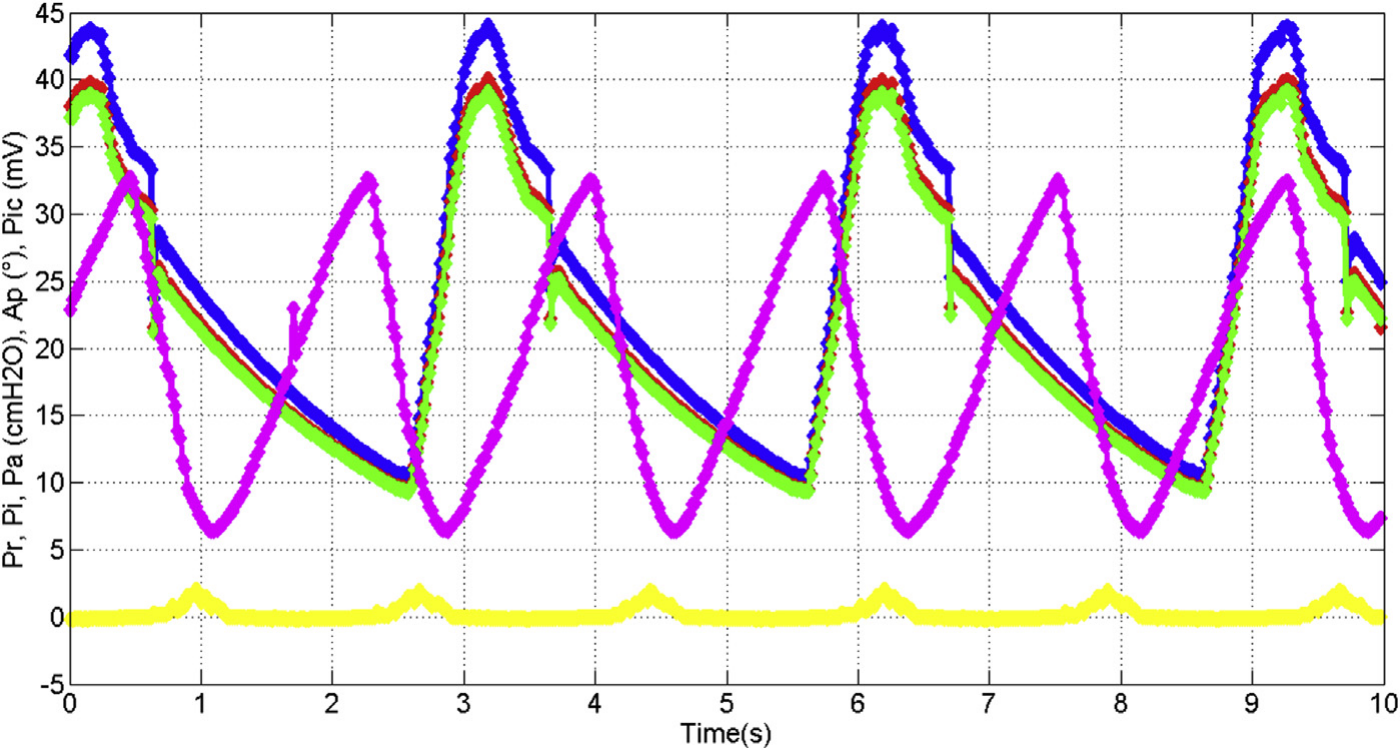
Pressure estimations.

In Figure 24, it is depicted the measured pressure as reference (curve in blue color), the identified pressure (curve in red color) and the adaptive-predictive pressure (curve in green color), moreover, it is shown the rotor angle displacement (curve in violet color) and the differential pressure (curve in yellow color) given by integrated circuit “IC”. Hence, the pressure estimation (because of adaptive-prediction) is obtained as a consequence of the rotor angle displacement and differential pressure, even though the out of phase half cycle approximately between the reference pressure with the estimated and identified curves. The standard error is 0.05 for the airflow, 0.07 for the volume and 0.09 for the pressure.

**Figure 25:**
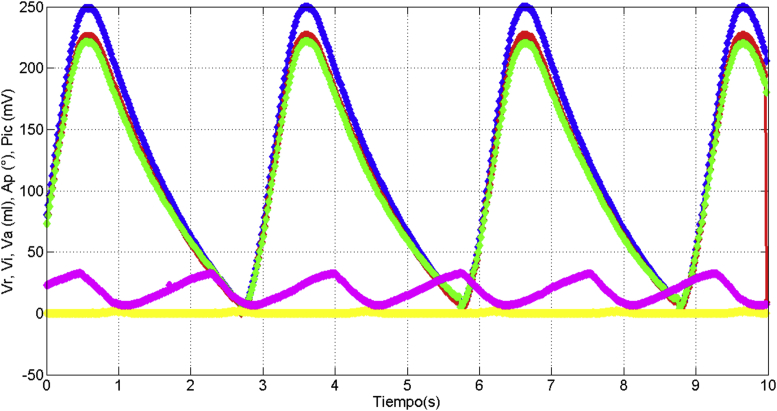
Volume estimations.

In Figure 25, it is depicted the measured volume as reference (curve in blue color), the identified volume (curve in red color) and the adaptive-predictive volume (curve in green color), moreover, it is shown the rotor angle displacement (curve in violet color) and the differential pressure (curve in yellow color) given by integrated circuit “IC”. Hence, the volume estimation (because of adaptive-prediction) is obtained as a consequence of the rotor angle displacement and differential pressure, even though the out of phase half cycle approximately between the reference volume with the estimated and identified curves. The standard error is 0.025 for the airflow, 0.012 for the volume and 0.05 for the pressure.

**Figure 26:**
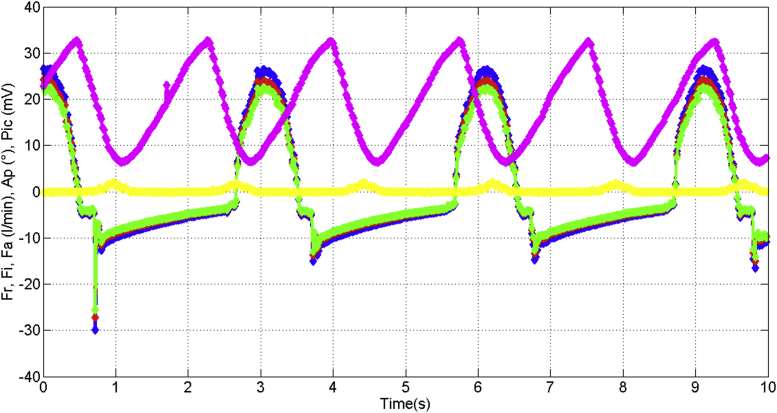
Air flow estimations.

In Figure 26, it is depicted the measured airflow as reference (curve in blue color), the identified airflow (curve in red color) and the adaptive-predictive airflow (curve in green color), moreover, it is shown the rotor angle displacement (curve in violet color) and the differential pressure (curve in yellow color) given by integrated circuit “IC”. Hence, the airflow estimation (because of adaptive-prediction) is obtained as a consequence of the rotor angle displacement and differential pressure, even though the out of phase half cycle approximately between the reference airflow with the estimated and identified curves. The standard error is 0.03 for the airflow, 0.02 for the volume and 0.04 for the pressure.

It is showed the following figure as outlook, the comparison between the differential pressure measurements due to obtain the airflow estimation with the airflow measured by a sensor based in nanostructures of Anodic Aluminum Oxide (AAO) are given in their faster response time (in comparison with the response time of the system) and robustness in front of disturbances. This measurement of airflow is showed through the yellow curve, the reference signal is shown by the green curve, the airflow that was measured by the sensor of the designed ventilator is given by the red curve and the blue curve in the measurement of an integrated circuit sensor. Therefore, it is possible to propose an outlook to analyze enhancement of the airflow measurement by sensors that were based in nanostructures.

**Figure 27:**
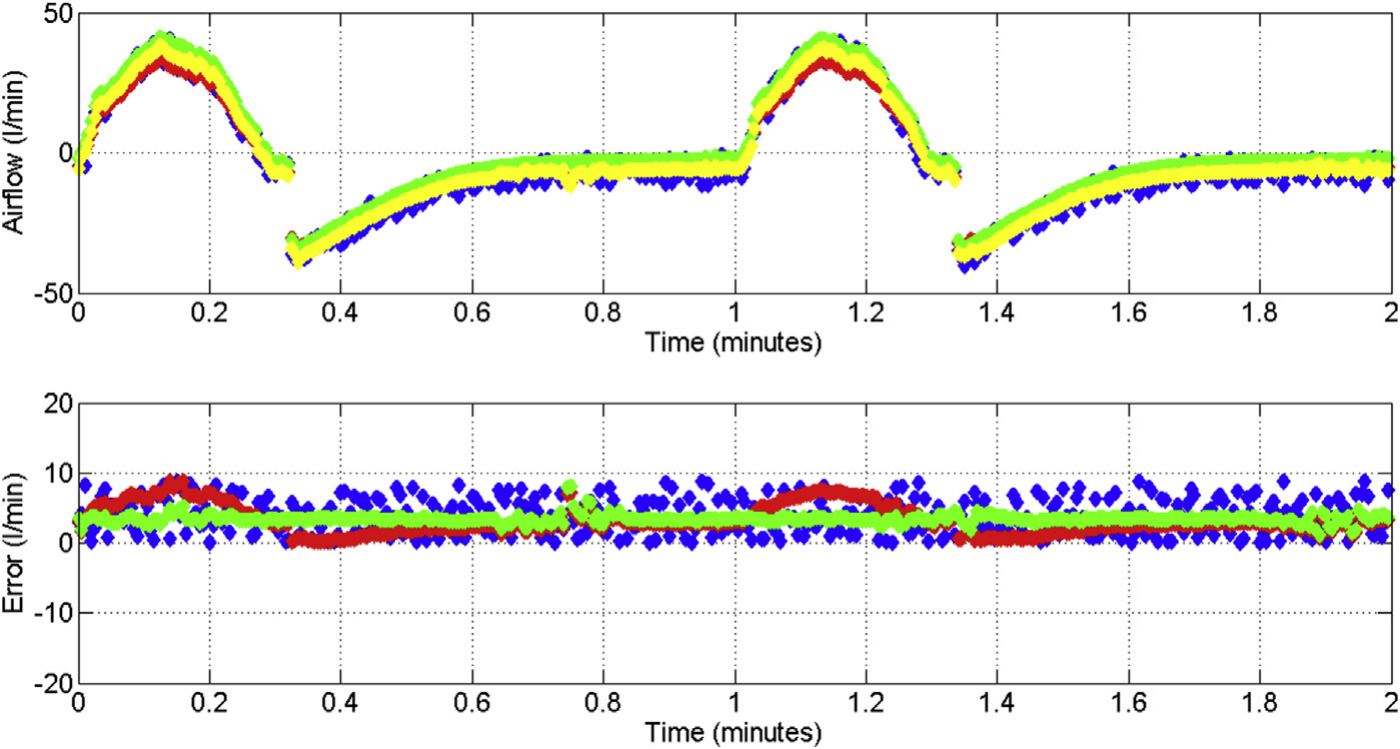
Results comparison from differential pressure sensors: based on integrated circuit sensor, air pressure sensor and air pressure sensor based on nanostructures.

Furthermore, in Figure 27, it is shown the error curves between the reference and data that was obtained by the sensors, which were used for the analysis. The blue color curve shows the error between the reference signal with an integral circuit (IC) from ARDUINO microcontrollers, that proportionate differential pressure according to achieve through the mathematical analysis that was described in the paragraphs above. The red color curve shows the error between the reference signal with the designed sensor transducer (ST). The ST was characterized, compensated and calibrated by FLUKE company sensors from the Biomedical Engineering Laboratory at PUCP. Finally, the green color curve shows the error between the reference signal with the airflow sensor based on nanostructures of Anodic Aluminum Oxide (AAO), which is reaching proposal sensor that was designed in the Researching Laboratories to enhance the optimization the mechatronic systems.

Therefore, the error from the IC has not stable performance and its average value that is around 5 l/min, the error from the designed ST reduced the average value at 3.2 l/min and 0.03 as standard error, but it keeps periodical overshoots which is a disadvantage for control tasks, the error from the sensor based in nanostructures of AAO reduces the average value to 3 l/min, that is not so much significant reduction in comparison to the ST. However, it has not periodical overshoots during the measurements because of the robustness and short response time of this kind of sensors.

For the mechanical ventilator that was designed in this work, it was chosen to use the ST due to it was very practical to find components to design it. This is a good advantage under pandemic time owing to markets tend to be in emergency social restrictions, moreover the ST had to be calibrated, because of using in the designed mechanic ventilator. It was not chosen the sensor based on nanostructures due to it is under researching tasks to enhance controllability and stability of mechanical systems. Nevertheless, it is proposed as an outlook of this kind of ventilator, when it could need control tasks. The standard error is 0.011 for the pressure.

Hence, it is proposed an outlook to enhance sensing ventilation variables through robust and faster sensors based on new technologies such as nanostructures. However, many of them are under researching tasks yet, which need certification validations [10, 13].

## 5 Conclusions

OxygenIP.PE is a redesigned low-cost mechanical ventilator in order to satisfy supply, technical and ventilation monitoring requirements in Peru. Ventilation curves produced by the OxygenIP.PE maintain the same behavior and magnitude as the original design in spite of the use of different power sources and airbag resuscitators which are not available and would produce different mechanical ventilation curves since their different properties, capacity, geometry and dimensions. Components were upgraded to allow a longer lifetime and improvement of performance in terms of friction, vibration and noise reduction. This suggests OxygenIP.PE is able to be medically validated by the government, as the original, in an emergency context while there aren't traditional ventilators available and for short periods of time use under medical staff supervision.

The control algorithm developed maintains a previous set rotation frequency of the system over time in spite of variations of system requirements due to its operation. Torque and rotation provided by the DC motor changes over time depending what the system needs, a high cam size for a high air volume increases the torque needed or the own transient nature of the ventilations curves changes the operation over time. In this sense, the control algorithm is able to provide a determined current calculated over time to control the response of the mechanical ventilation system OxygenIP.PE, which ensures a correct operation needed for a medical equipment.

Ventilation parameters monitoring were developed and installed in order to provide useful information to medical staff. The designed mathematical model is able to estimate and to identify the physical variables and geometrical parameters of the mechanical ventilator prepared by cams. A monitoring algorithm was designed to visualize the identified and estimated physical variables: “pressure, volume and airflow”. The mechanical ventilator, as a consequence of the mathematical model and monitoring algorithm designed, can proportionate the estimation of the “pressure, volume and airflow” from the correlation of the angular position of the oscillating follower pivot. This is measured over time by the sensors implemented. Furthermore, by measuring differential pressure, it was possible to estimate in a better way the volume and airflow.

Finally, OxygenIP.PE is still a low-cost equipment, sturdier and more sophisticated than the original, able to be fabricated in mass in Peru regardless of import availability of special electronic components.

## Declarations

### Author contribution statement

J. Alan Calderón Ch.: Conceived and designed the experiments; Performed the experiments; Analyzed and interpreted the data; Wrote the paper.

Carlos Rincón: Conceived and designed the experiments; Analyzed and interpreted the data; Contributed reagents, materials, analysis tools or data; Wrote the paper.

Martin Agreda: Contributed reagents, materials, analysis tools or data.

Juan José Jiménez de Cisneros: Analyzed and interpreted the data; Contributed reagents, materials, analysis tools or data.

### Funding statement

This work was supported by the 10.13039/501100010751CONCYTEC-FONDECYT en el marco del concurso "PROYECTOS ESPECIALES: Modalidad - Escalamiento de proyectos COVID-19" [74169]. a Motores Diesel Andinos S. A. -MODASA [74169], a Pontificia Universidad Catolica del Peru [74169], y a Protofy.XYZ S.L. [74169].

### Data availability statement

Data included in article/supp. material/referenced in article.

### Declaration of interests statement

The authors declare no conflict of interest.

### Additional information

No additional information is available for this paper.
